# “I heard the heartbeat”- Psychophysiological and Sociocultural Determinants in Pseudocyesis and Delusion of Pregnancy

**DOI:** 10.1192/j.eurpsy.2022.608

**Published:** 2022-09-01

**Authors:** A. Akira, A. Deka, D. Banayan

**Affiliations:** Rush University Medical Center, Psychiatry, Chicago, United States of America

**Keywords:** Pregnancy, pseudocyesis, delusion of pregnancy, somatic symptom disorder

## Abstract

**Introduction:**

Pseudocyesis and Delusion of Pregnancy are often conflated. Both presentations are associated with false beliefs of pregnancy in patients who are not pregnant. Pseudocyesis is associated with physiological changes of pregnancy such as amenorrhea, galactorrhea, abdominal distention, and hyperprolactinemia. Delusion of Pregnancy is not associated with physiological signs/changes. We describe a case to demonstrate the phenomenological and physiological differences between these entities and how these influence treatment considerations.

**Objectives:**

1.Phenomenology of Pseudocyesis vs Delusion of Pregnancy 2.Elucidate the physiological underpinnings of both 3.Treatment considerations

**Methods:**

Comprehensive literature review following a 29-year-old-female with no known psychiatric history presenting to the emergency department with mixed complaints of twin-pregnancy, menorrhagia, and concern for threatened abortion. Psychiatry was consulted for decisional capacity to leave against-medical-advice due to concerns for ectopic pregnancy. Patient reported a recent ultrasound with fetal heartbeat and sensation of fetal “kicks”. She was concerned the menorrhagia was threatening her pregnancy. The patient appeared irritable, paranoid, endorsed ideas of reference and a fixed false belief that she was pregnant with twins, despite quantitative HCG, abdominal and transvaginal ultrasounds being negative. On examination, while there was vaginal bleeding, there were no stigmata of pregnancy.

**Results:**

Diagnosis- Delusion of Pregnancy.

**Conclusions:**

Delusion of Pregnancy have been associated with polythematic content. Pseudocyesis may be confounded by conditions such as abdominal neoplasms, leiomyoma, and endocrinologic changes (eg- hyperprolactinemia). Potent D2R antagonists carry increased risk of hyperprolactinemia and subsequent galactorrhea which may paradoxically exacerbate misattributions of pregnancy. Careful consideration of psychotropic choice is therefore needed in the management of these conditions.

**Disclosure:**

No significant relationships.

